# Integrative analyses of morpho-physiological, biochemical, and transcriptomic reveal the seedling growth response of *Pinus yunnanensis* to nitrogen and phosphorus fertilization

**DOI:** 10.3389/fpls.2024.1405638

**Published:** 2025-01-27

**Authors:** Junfei Xu, Xiyan Li, Shi Chen, Lin Chen, Junrong Tang, Peizhen Chen, Nianhui Cai, Yulan Xu

**Affiliations:** ^1^ Key Laboratory of National Forestry and Grassland Administration on Biodiversity Conservation in Southwest China, Southwest Forestry University, Kunming, China; ^2^ The Key Laboratory of Forest Resources Conservation and Utilization in the Southwest Mountains of China Ministry of Education, Southwest Forestry University, Kunming, China; ^3^ Technology Promotion Department, Kunming Station of Forestry and Grassland Science and Technology Promotion, Kunming, China

**Keywords:** *Pinus yunnanensis*, proportioning fertilization, growth, biomass, chlorophyll, nutrient element, transcriptome

## Abstract

Appropriate nitrogen (N) and phosphorus (P) fertilization is critical for plant growth and production. *Pinus yunnanensis*, a silvicultural tree in southwestern China, faces economic and ecological limitations due to nutrient deficiency in the soils in its distribution areas. The slow growth of this species during the seedling stage exacerbates these problems. Therefore, it is important to realize the regulating effects of N and P proportioning fertilization on seedling growth to enhance nutrient-use efficiency. In this study, variations in morphological, physiological, and biochemical characteristics of seedlings were analyzed under nine treatments of NP proportioning in an open nursery using a regression design. Growth in height and basal diameter increased and showed an approximate tendency in all treatments. The maximum biomass accumulation was observed at 480 d after treatment in roots of T5 (14.714 g) (application N 0.4 g·per^−1^ and P 3 g·per^−1^), stems of T5 (12.654 g), leaves of T9 (24.261 g) (application N 0.8 g·per^−1^ and P 6 g·per^−1^), aboveground parts of T9 (35.402 g) and individuals of T5 (49 g). The total chlorophyll content peaked in the leaves at 120 d and was correlated with the peak levels of N, P, and K in leaves. The content and reserves of nutrient elements in the organs of seedlings subjected to NP proportioning were significantly higher than those in unfertilized seedlings. Analysis of nutrient utilization efficiency revealed that T5 demonstrated superior seedling growth performance. Appropriate fertilization dosage of N and P for *P. yunnanensis* seedlings in this study was 0.32 g·per^−1^–0.58 g·per^−1^ and 3.02 g·per^−1^–4.95 g·per^−1^ respectively, using path analysis and regression equation. Transcriptomic sequencing revealed that there were 2,301 DEGs between T5 and T1 (control), which were involved in the uptake and assimilation of nutrients, biosynthesis of phytohormones and secondary metabolites, and photosynthesis. Additionally, the abundance of genes involved in cell division and proliferation, cellulose biosynthesis, and cell wall extension were dramatically upregulated, which potentially correlated with enhanced seedling growth. In conclusion, this study provides comprehensive information on the response of seedlings to varying proportions of N and P and may promote the growth of *P. yunnanensis* seedlings by optimizing the proportion of N and P in fertilizers.

## 1 Introduction

*Pinus yunnanensis*, one of the main silvicultural conifers with strong adaptability and vigorous growth in southwestern China, plays an important role in forest production and sustains regional ecosystem stability (Gao et al., 2023). However, most forest stands with inferior productivity are caused by poor and/or inappropriate fertilization. This results in slow growth during the seedling stage, which significantly limits their benefits (Daubresse et al., 2010; Ham et al., 2018). Especially in the habitat of *P. yunnanensis*, the soil in the distribution areas is extremely deficient in nitrogen (N) and phosphorus (P) but rich in potassium (K) (Jin and Peng, 2004). The productivity of crops and plantations depends heavily on fertilization. Previous studies have shown that exogenous nutrient application is an important measure for improving seedling growth and shortening the breeding cycle (Xu et al., 2012). Therefore, studying fertilization to promote seedling growth and increase *P. yunnanensis* productivity is very important.

N and P are essential macronutrients absorbed by roots and are reserved in the aboveground parts of plants. They are involved in the biosynthesis and metabolism of proteins, amino acids, and carbohydrates, thereby improving root system development, plant growth, and agroforestry productivity (Razaq et al., 2017). In recent decades, many studies have focused on the biological processes of uptake, translocation, assimilation, and signaling of nutrient elements in *Arabidopsis*, rice, tobacco, maize, and poplar (Daubresse et al., 2010; Xu et al., 2012; Wang et al., 2018; Ham et al., 2018; Han et al., 2020). Several protein families are involved in the uptake and translocation of N and P, including the *Nitrate Transporter 1/Peptide Transporter Family* (NPF), *Nitrate Transporter 2* (NRT2), *Chloride Channel* (CLC), *Ammonium Transporter* (AMT)and *Phosphorus Transporter* (PHT) (Ayadi et al., 2015; Ham et al., 2018; Han et al., 2020). As sessile organisms, plant root systems are capable of absorbing N and P to expand their habitats and achieve reproduction. Root architecture is modified in response to N, particularly in secondary roots (King et al.,1996; Zhang et al., 2019). For example, plants such as *Arabidopsis*, rice, maize, beans, and members of the *Proteaceae* family have the potential to substantially increase secondary root growth and underground biomass in response to low soil fertility (Francisco et al., 2015; Xin et al., 2021). In poplars, NRT2;7 plays a key role in nitrate transport during leaf development (Zhao et al., 2021). *PdePHT1;9* positively regulates Pi translocation from roots to shoots and increases shoot Pi content under both normal and low P applications (Yang et al., 2022).

The fixed carbon and carbohydrates supplied by photosynthesis are also necessary to improve the activity of the roots and aerial parts (Xu et al., 2012). The remobilization of N from roots to shoots and leaves improves cell division and photosynthetic capacity (Li et al., 2018). Previous reports have shown that photochemical efficiency is positively correlated with the leaf N content. Through fertilization, the chlorophyll content and net photosynthetic rate of *Machilus pauhoi* seedlings increased by an average of 40.115% and 150.17%, respectively (Hu et al., 2015). Transcriptome and proteome analyses revealed that nitrate and ammonium increased photosynthetic efficiency by regulating glnA and rbcS in *Phoebe bournei* (Wang et al., 2024). Genes related to the cytochrome b6f complex and glycolysis were highly expressed, and the large subunit RuBisCO was also upregulated by western blotting, indicating that fertilization may effectively enhance leaf photosynthetic capacity in *Cunninghamia lanceolata* (Zhang et al., 2016). These processes also involve a complex phytohormone signaling network. For instance, auxins mediate N signals from shoots to roots and nitrate can directly increase the expression of AFB3 (an auxin receptor) (Kiba et al., 2011). Nitrate also induces the expression of IPT3, a key biosynthesis gene of cytokinin (CK) (Takei et al., 2004). Studies have shown that CK improves the expression of NRTs in shoots, while repressing their expression in roots, suggesting that CK plays a role in the response to N saturation (Sakakibara et al., 2006).

Seedlings have specific requirements at different developmental stages, owing to their biological characteristics and cultivation methods (Zhang et al., 2008). However, inappropriate fertilization hinders seedling growth and may cause resource wastage or environmental pollution (Liu et al., 2016). Therefore, it is necessary to conduct research on the optimal nutrient dosage and proportion of forestation species. A previous study revealed that NP proportioning can regulate nutrient transformation in soil, which not only meets the nutrient needs for seedling growth but also greatly improves the uptake and utilization efficiency of fertilizer (Wang et al., 2016). A medium level of N and P fertilizers increased the net photosynthetic rate (by 43.48%) and water use efficiency (by 127.75%) of *Phoebe hui* seedlings, with biomass accumulation being 46.67% higher than that of the control (Zhou et al., 2021). Similarly, the height, basal diameter, accumulation of biomass, nutrients, and chlorophyll concentration of *P. massoniana* and *Tilia amurensis* seedlings increased significantly under a medium proportion of NP (Yang et al., 2021; Luo et al., 2021). We speculate that applying an appropriate proportion of NP fertilizer to the soil may contribute to the growth and production of *P. yunnanensis* seedlings.

However, the effects of different proportions of NP fertilization on the growth (phenotypes), physiological responses, and biochemical characteristics of *P. yunnanensis* seedlings have not been studied. The optimal dosage and proportion of NP for promoting seedling growth remains unclear. Here, we tested different proportions of N and P to investigate the morphological, physiological, and biochemical characteristics of seedlings in response to fertilization. Consequently, the optimal fertilization dosage and proportion of NP required for seedling growth were established. Differences in transcript abundance related to cell proliferation, cell wall formation and extension, and cellulose biosynthesis may be crucial factors contributing to seedling changes. The objective of this study was to provide the foundational information needed to adopt strategies employed by seedlings to cope with various nutrients. Moreover, this information could also be used as guidance to improve nutrient use efficiency and further enhance productivity by increasing afforestation efficiency and shortening breeding cycles.

## 2 Materials and methods

### 2.1 Plant materials and experimental design

Seeds from half-sibling families were collected from a clonal seed orchard in Midu County, Yunnan Province, China. The experimental field was set up in an open nursery garden located at the Southwest Forestry University, Kunming, China (**Supplementary Figure S1**). The region’s climate is characterized by an average temperature of 14.7 °C, an average annual precipitation of 942.5 mm, and an altitude of approximately 1,945 m. The experiment workflow is illustrated in **Supplementary Figure S2**. In January 2020, the seeds were treated with 0.5% KMnO_4_ solution for 30 minutes (min) and then soaked in water for 48 hours (h) to promote germination. They were further cultured in mixed substrates of humus and krasnozem at a 3:1 ratio. The pH range of the substrates ranged between 6.0 and 6.2. The contents of total nitrogen (TN), total phosphorus (TP), and total potassium (TK) in the cultural substrates were 1.15 g·kg^−1^, 0.89 g·kg^−1^, and 9.03 g·kg^−1^, respectively. Seedlings were not treated with any type of fertilization until the experiment was initiated. According to a previous study by Cai et al. (2019), to determine the fertilization dosage, all seedlings were fertilized concurrently with urea (with a nitrogen content of 46%) and superphosphate (with a phosphate content of 12%) at the same time as the field planting. The experimental design employed a regression model that included two nutrient elements (N and P) and three concentration levels (high, medium, and low fertilizer dosage), resulting in nine treatments, which were combinations of N and P at these three levels. Nitrogen fertilizer levels were 0 g per seedling (g·per^−1^) (N1), 0.4 g·per^−1^ (N2), and 0.8 g·per^−1^ (N3). Phosphorus fertilizer levels were 0 g·per^−1^ (P1), 3 g·per^−1^ (P2), and 6 g·per^−1^ (P3) (**Supplementary Table S1**). In August 2020, 1,620 uniformly sized seedlings were planted in pots (diameter: 16 cm, height: 13.5 cm) and arranged in a randomized complete block design with three replications, with 60 seedlings per replicate for each treatment. After planting, the seedlings were regularly weeded and irrigated three times a week to promote growth and minimize variation among the seedlings.

### 2.2 Morphological measurements

After planting, all seedlings were measured monthly for their height using a ruler and their basal diameter using a digital Vernier caliper, accurate to 1 mm and 0.01 mm, respectively. To obtain further information about seedling growth and development, we randomly selected three seedlings from each treatment and replicated for the determination of biomass, chlorophyll content, and nutrient content at 120 days (d), 210 d, 300 d, 390 d, and 480 d after planting (n = 9 for each time point). During harvesting, all samples were carefully taken from a single seedling in each treatment group, following the procedure described by Guo et al. (2008). The fresh masses of the roots, stems, and leaves of the tested samples were measured and recorded using an electronic balance. Additionally, the roots were scanned using an Epson scanner and morphological characteristics were obtained using WinRHIZO. After that, the tested samples were dried in an oven at 105 °C for 30 minutes (min), and then at 80 °C until they reached a constant weight, which were used to measure the dry biomass (accurate to 0.0001 g). Seedling biomass, consisting of roots, stems, and leaves, was determined. Based on these measurements, the aboveground biomass, individual biomass, and biomass distribution ratios of the units were calculated.

### 2.3 Physiological and biochemical analyses

Mature leaves from the same year were collected for the determination and analysis of chlorophyll pigment content. Their contents were detected using a spectrophotometer following a previously described method involving acetone extraction (Cai et al., 2019). To access the nutrient information of organs, the tested samples were crushed in a mill and subjected to the H_2_SO_4_–H_2_O_2_ heating digestion method to determine the contents of TN, TP, and TK in roots, stems, and leaves, as described previously (Bao, 2020). Briefly, dried samples were pulverized and sieved. Then, 0.2 g of the sample was then placed in a digestion tube with 1.5 mL ddH_2_O and concentrated sulfuric acid (5 mL). Hydrogen peroxide was added to the digestion tube and heated at 80°C, 160°C, 240°C, 280°C, and 340°C using a far-infrared digestion furnace until the solution turned colorless or clear. The boiled solution was diluted with ddH_2_O to a final volume of 50 mL. Nutrient element reserves were obtained by multiplying the nutrient element content of the organs by their corresponding organ biomass.

### 2.4 RNA extraction and transcriptome sequencing

Sequencing analysis of the transcriptome was performed by PANOXIX Biomedical Tech Co. Ltd. (Suzhou, China). Approximately 120 d after the initiation of fertilizer treatment, based on the results of phenotypic, physiological, and biochemical analyses conducted on nine treatments, seedling samples from T1 and T5 were chosen for transcriptome sequencing. The seedling needles were isolated and ground in liquid nitrogen. The RNAprep pure plant kit (TianGen, China) was used to extract total RNA from the samples (three replicates per treatment), following the manufacturer’s protocol. The mRNA was enriched using Oligo(dT) beads, and the purified mRNA was cut into short fragments of approximately 300 bp. Reverse transcriptase was used to synthesize cDNA. All samples were sequenced using the Illumina NovaSeq 6000 (Illumina, USA) in paired-end mode after the libraries were constructed and qualified (Niu et al., 2019). After filtering and quality control, the raw data were mapped to the *P. taeda* reference genome (Pita v2.01, https://treegenesdb.org) using the Bowtie and BWA software. The assembled transcripts were spliced using clean data, and their expression was calculated using FPKM (Bray et al., 2016). All raw RNA-seq data were deposited in the SRA under the Bioproject PRJNA1086820 (https://www.ncbi.nlm.nih.gov/bioproject/PRJNA1086820/).

### 2.5 Transcriptomic analysis

To obtain functional gene information, the assembled sequences were aligned with sequences from public databases, including Gene Ontology (GO), Kyoto Encyclopedia of Genes and Genomes (KEGG), Protein family (Pfam), orthology relationships, functional annotation, and gene evolutionary histories (eggnog), Swiss-Prot (a manually annotated and reviewed protein sequence database), and the NCBI non-redundant protein sequence database (Nr), using BLAST (Altschul et al., 1997), as previously described (Li et al., 2016). Differentially expressed genes (DEGs) between treatments were identified using the DESeq2 R package (version 3.10; Love et al., 2014). Using previous methods with a threshold for values of p <0.05, and |log_2_ (FC)| ≥1, it is considered to be significantly differentially expressed (Benjamini and Yekutieli, 2001).

### 2.6 Verification by RT-qPCR

Eighteen genes were selected for verification using quantitative real-time PCR (RT-qPCR). Total RNA was extracted from T1 and T5 needles. cDNA was synthesized using a HiScript 1st Strand cDNA Synthesis Kit (Vazyme, China). The expression levels of the target genes were detected using the Taq Pro Universal SYBR qPCR Master Mix (Vazyme, China) on the Rotor-Gene Q 5plex Platform (QIAGEN, Germany). All primers were designed using Oligo 7.0, and *Tubulin* (*PITA_49533*, encoding a Tubulin alpha-1 chain protein) was used as a reference gene (**Supplementary Table S2**). Each reaction consisted of 10 μl SYBR mix, 0.4 μl of each primer solution, and 100 ng of cDNA in a final volume of 20 μl. The reaction mixtures were prepared according to the kit protocol as follows: 95 °C for 2 min, 40 cycles of 95 °C for 5 seconds (s), 60 °C for 5 s, and 72 °C for 25 s. Each sample contained three biological replicates. Relative expression levels were calculated using the 2^−ΔΔCT^ method (Livak and Schmittgen, 2001).

### 2.7 Statistical analyses

The results were subjected to analysis of variance (ANOVA) using Excel 2016 and SPSS 27.0, and the data are presented as mean ± standard error (SE). Different letters indicate statistically significant differences, as determined by one-way ANOVA, followed by Duncan’s method for multiple comparisons (P <0.05). Pearson’s correlation analysis was used to examine the correlation between different databases, and path analysis was used to investigate the effects of various factors on seedling growth (Cai et al., 2019). GraphPad prism 7.5 was used to draw graphs.

## 3 Results

### 3.1 Effects of fertilization on seedling growth and biomass

**Figure 1:**
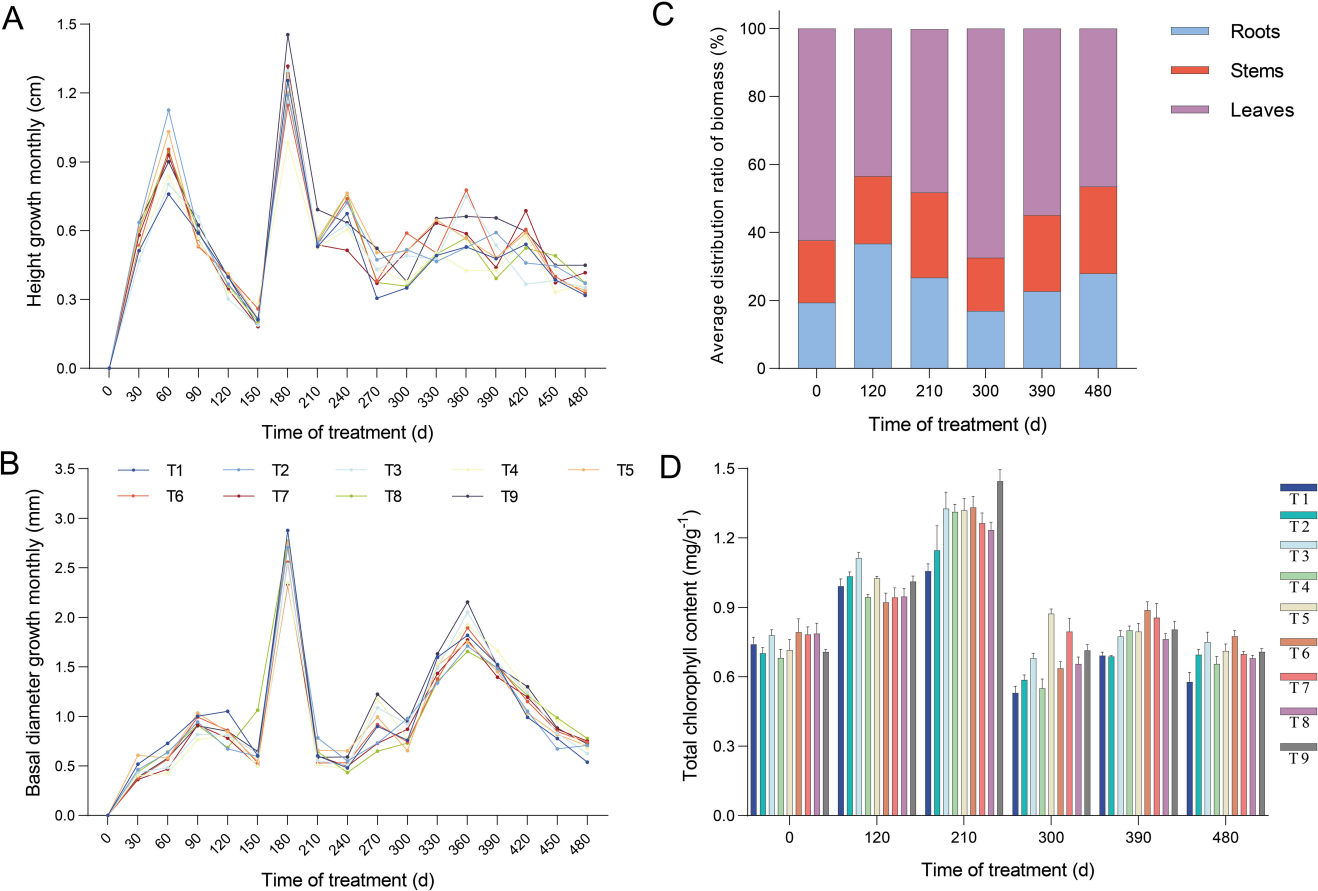
Changes in characteristics of seedlings growth and development under different proportions of N and P application. **(A)** Monthly variation in the height growth of seedlings at different fertilization levels. Accurate to 1 mm. **(B)** Monthly variation in the basal diameter growth of seedlings at different fertilization levels. Accurate to 0.01 mm. **(C)** The response of the average biomass distribution ratio of organs during seedlings growth. **(D)** The effect of fertilization on total chlorophyll content. Error bars stand for the mean ± standard error (SE) of three replicates. Statistically significant differences and complete data are presented in the **Supplementary Tables S3**, **S5**, and **S6**.

**Figure 2:**
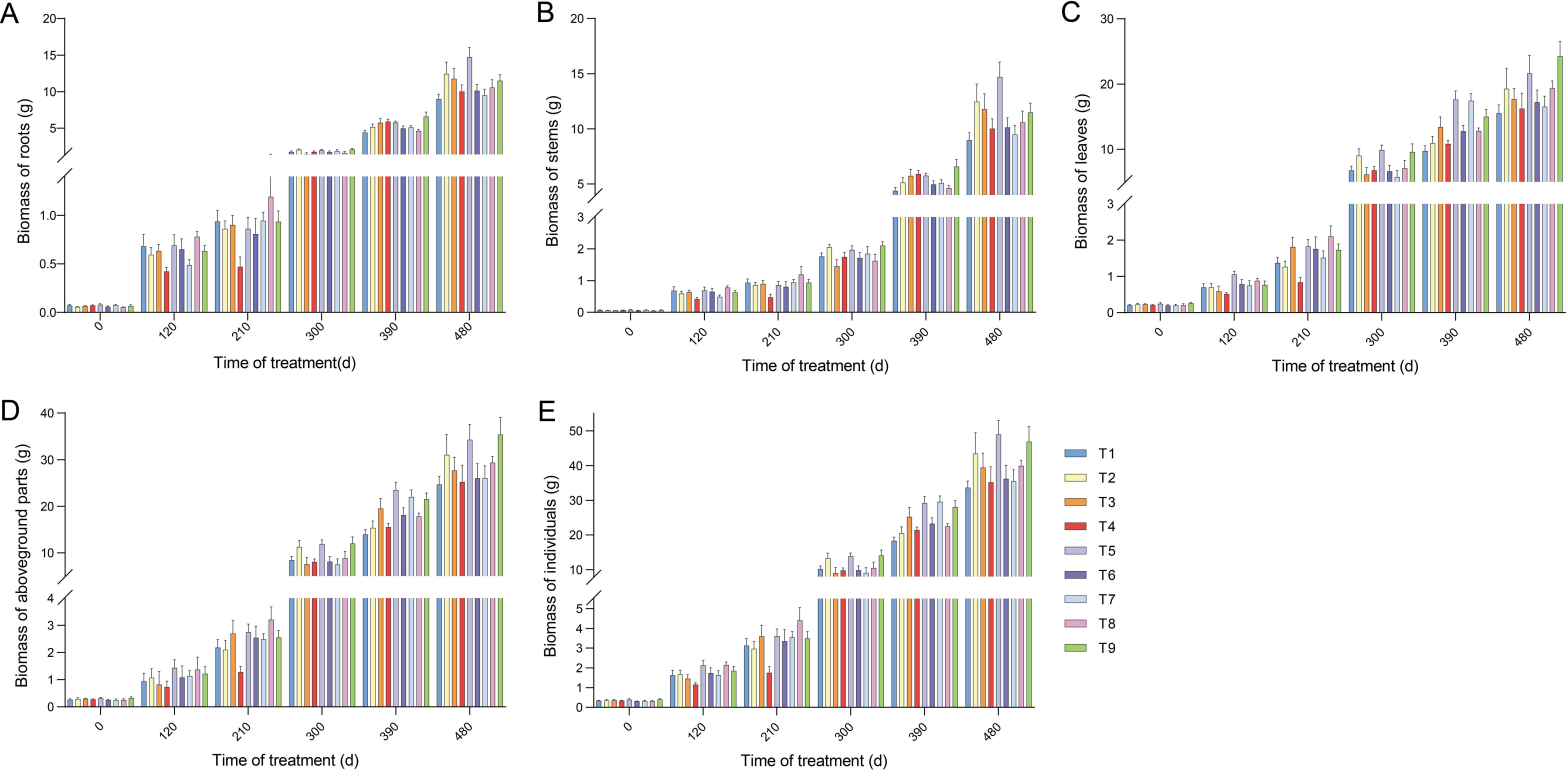
Changes in biomass accumulation in the organs and units of seedlings under different proportions of N and P application. **(A)** Biomass accumulation of roots. **(B)** Biomass accumulation of stems. **(C)** Biomass accumulation of leaves. **(D)** Biomass accumulation of aboveground parts. **(E)** Biomass accumulation of individuals. Error bars stand for the mean ± SE of three replicates (N = 9). Statistically significant differences and complete data are presented in the **Supplementary Table S4**.

To investigate the effect of N and P application on seedlings growth, we measured the height, basal diameter, root morphology, and biomass of *P. yunnanensis* seedlings in the nine treatments. All treatments showed a significant increase in height at 60 d and 180 d after fertilization, with a greater enhancement in basal diameter observed at 90 d, 180 d, and 360 d (**Figures 1A, B**). The growth patterns of height and basal diameter across all treatments exhibited an approximate tendency, indicating that fertilization did not alter the growth trend of seedlings. At 480 d, the height of T9 and the basal diameter of T8 were greater than those of the other treatments (**Supplementary Table S3**). The morphology and spatial distribution of plant roots determine the efficiency of plants in acquiring water and nutrients. To characterize root growth, we investigated root morphology and biomass during the fertilization period. The root biomass in all treatments showed a significant increase at 120 d, with a nearly 10-fold increase compared to that at 0 d. At 480 d, T5 exhibited greater biomass than the other treatments, with an increase of 63.8% compared to T1 (**Figure 2A**; **Supplementary Table S4**). However, root scanning revealed no significant differences in root growth among all treatments, and no significant correlation was found between height and basal diameter (data not shown).

We then investigated the impact of the nine treatments on the seedling biomass. Compared with T1, the application of N and P increased the aboveground biomass of the seedlings. Similar to the results observed for the roots, T5 and T9 showed higher biomass accumulation. This increase also included growth in both stem and leaf biomass (**Figures 2B–D**; **Supplementary Table S4**). Notably, the biomass accumulation rate of leaves was higher than that of roots and stems at 210 d after fertilization. The biomass accumulation of individuals among all treatments increased rapidly over time with fertilization and accelerated significantly after 120 d (**Figure 2E**). In summary, the maximum biomass accumulation in all treatments was observed in the roots of T5, stems of T5, leaves of T9, aboveground parts of T9, and individuals of T5 (**Figures 2A–E**). Furthermore, the biomass distribution ratios in the organs and units were differentially influenced by fertilization (**Figure 1C**; **Supplementary Table S5**). The distribution ratio of root biomass, averaging 36.71% across all treatments, was higher at 120 d after fertilization than at other times. The aboveground biomass was also reduced to a minimum at 120 d. The leaves and aboveground parts peaked at 300 d after fertilization, averaging 67.51 % and 83.27%, respectively, whereas the stems reached their peak (averaging 25.48%) at 480 d. Taken together, these results suggest that seedling biomass was increased by NP proportion fertilization, with T5 exhibiting a more pronounced increase.

### 3.2 Chlorophyll content and nutrient elements in response to fertilization

To further confirm whether the observed morphological variations were due to fertilization with different N and P proportions, the study examined the content of total chlorophyll and nutrient elements (TN, TP, and TK) in the nine different treatments was measured. We found that the total chlorophyll content across all treatments increased at 120 d after fertilization. The chlorophyll content when P was applied alone was significantly higher than that in the other treatments (**Figure 1D**; **Supplementary Table S6**). Each treatment reached its maximum content at 210 d, among which the content of T9 was significantly higher than that of the other treatments, with 1.37-fold higher than that of T1. At 480 d, the content of T2–T9 increased by 13.3%–34.1% relative to T1. The total chlorophyll contents of T3, T9, T5, T6, and T6 were the highest from 120 d to 480 d after fertilization. The total chlorophyll content of the seedlings was significantly improved by NP proportioning. The effect of P on promoting chlorophyll synthesis is stronger than that of N.

In this study, different proportions of N and P significantly affected the contents of TN, TP, and TK in the roots, stems, and leaves (**Supplementary Table S7**). NP proportion fertilization greatly increased the nutrient content of seedling organs in the early stages. The contents of TN, TP, and TK in roots and leaves peaked from 120 d to 210 d after fertilization, which coincided with the timing of maximum total chlorophyll content, root biomass accumulation, and distribution proportion of root biomass. Meanwhile, the contents of TN, TP, and TK in the stems peaked at 210 d, 390 d, and 300 d after fertilization, respectively. As time progressed, fertilizer efficiency diminished, and the nutrient content in the organs appeared to decrease to some degree. These findings suggest that NP proportion fertilization promotes the uptake and translocation of nutrients from the roots to the aerial parts. Moreover, the nutrient element reserves of seedlings significantly increased following fertilization, with T5 exhibiting the highest TN and TP levels in the roots, as well as TN, TP and TK in the stems. T9 showed the highest TK in the roots and TN, TP, and TK in the leaves (**Supplementary Table S8**). Compared with the unfertilized seedlings, the nutrient element reserves in seedling organs treated with NP proportioning fertilization, particularly in T5 and T9, were significantly elevated. Leaf P reserve was primarily influenced by N, while the reserves of nutrients in other organs was primarily affected by P. This finding is consistent with the effects of NP proportion fertilization on seedling height, basal diameter, biomass, and total chlorophyll content. Furthermore, our analysis of nutrient utilization efficiency revealed that the roots, stems, and leaves of T5 exhibited higher N and P utilization than the other treatments (**Supplementary Table S9**).

### 3.3 Correlation between measured indicators and seedlings growth

NP proportion fertilization influenced the vegetative, physiological, and biochemical characteristics of seedlings, particularly in T5 and T9. A significant correlation was observed between morphological and physiological–biochemical changes, as determined by path analysis (**Supplementary Table S10**). Seedling height was significantly correlated with total chlorophyll content, stem biomass, leaf biomass, P reserves, and K content in the leaves. The basal diameter of the seedlings was significantly correlated with the N reserves in the leaves and stems. Subsequently, based on the results of the path analysis, we constructed binary and quadratic regression equations. The appropriate fertilization dosage of N in this study was 0.32 g·per^−1^–0.58 g·per^−1^, while that of P was 3.02 g·per^−1^–4.95 g·per^−1^. The optimal proportions of N and P ranged from 1:7.914 to 1:9.438, respectively (**Table 1**). In conclusion, these results suggest that the optimal treatment for this study was T5 (N2P2), which represents a medium proportion of N and P and likely led to better vegetative performance in *P. yunnanensis* seedlings.

**Table 1:** Fertilization dosage for the *P. yunnanensis* seedling growth.

Index with positive effect on seedling growth	Fertilization dosage		NP ratio
	N (g·per^−1^)^1^	P (g·per^−1^)	
Leaf biomass	0.54	4.65	1:8.611
Stem biomass	0.54	4.45	1:8.241
Total chlorophyll	0.52	4.66	1:8.962
Leaf K content	0.32	3.02	1:9.438
Stem N reserves	0.47	3.62	1:7.702
Leaf N reserves	0.58	4.59	1:7.914
Leaf P reserves	0.56	4.95	1:8.839
Leaf K reserves	0.55	4.16	1:7.564

^1^Note: the fertilization dosage applied per seedling (gram·per seedling).

### 3.4 Transcriptomic response to NP fertilization

**Figure 3:**
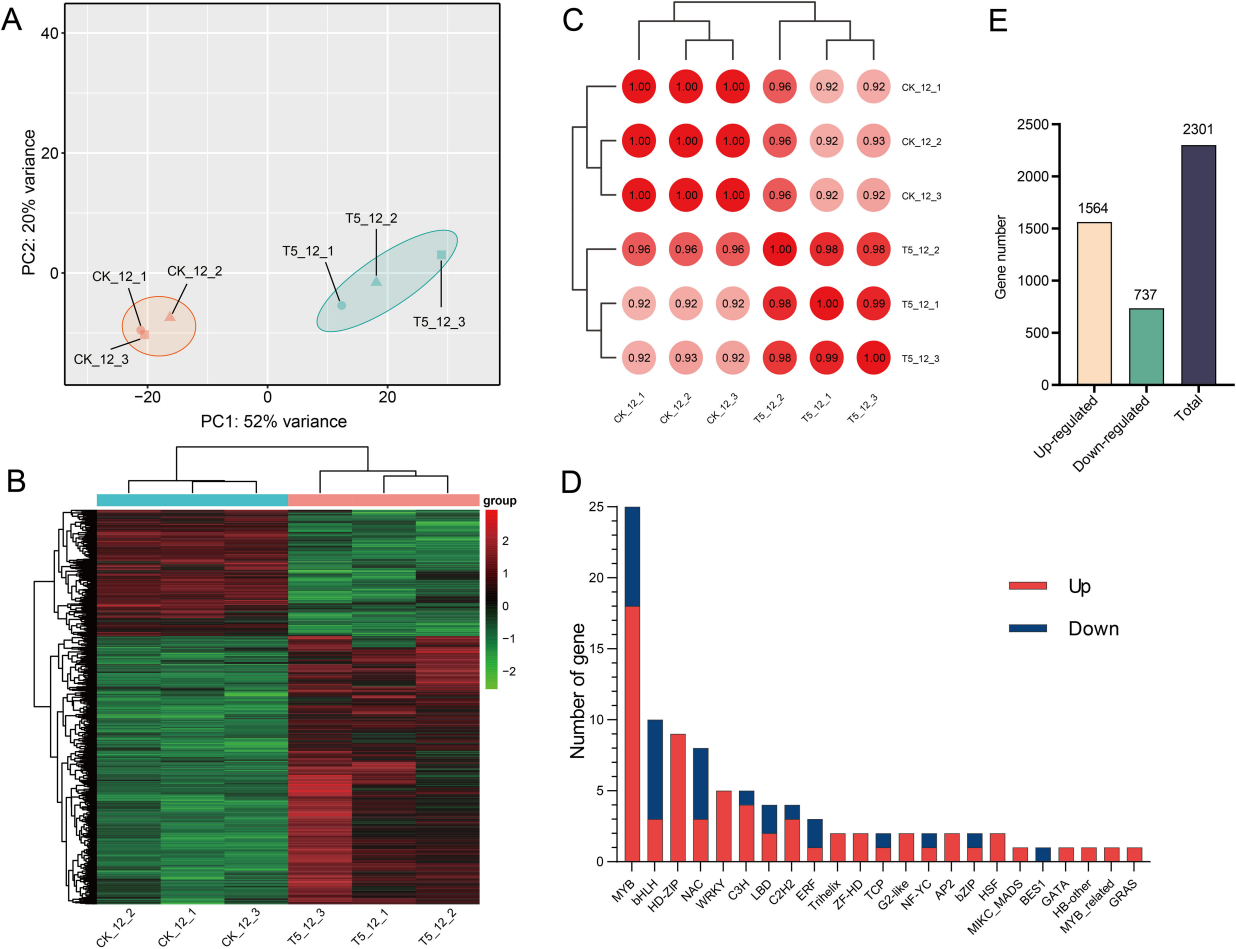
The transcriptome analysis of T1 and T5 seedlings after 120 d of medium proportion of N and P fertilization. **(A)** PCA of the treatment groups. **(B)** Heat map of DEGs from samples. Red represents highly expressed genes, green represents low-expression genes. **(C)** Pearson’s correlation coefficient of genes between samples. **(D)** The number of transcription factors in T5_12 (T5) vs CK_12 (T1) **(E)** The number of DEGs among treatments. Color scale represents the gene expression level.

Based on the aforementioned investigation, it was found that the growth of seedlings was significantly facilitated by NP proportioning. Samples of T5 at 120 d after fertilization (T5_12) were selected, and transcriptomic analysis was conducted to further monitor and comprehend the molecular changes induced by N and P on seedling growth. High-quality clean data were obtained for each tested sample, with >75% and >86% clean reads being total and uniquely mapped to the *P. taeda* reference genome, respectively (**Supplementary Table S11**). A total of 14,240 genes were detected to be expressed in at least one sample [FPKM>1]. PCA and cluster analysis showed distinct differences in expressional among all samples across the two components (**Figures 3A, B**). Pearson’s correlation coefficient analysis showed that the transcriptomic data were highly reproducible (≥0.92) across the samples (**Figure 3C**).

**Figure 4:**
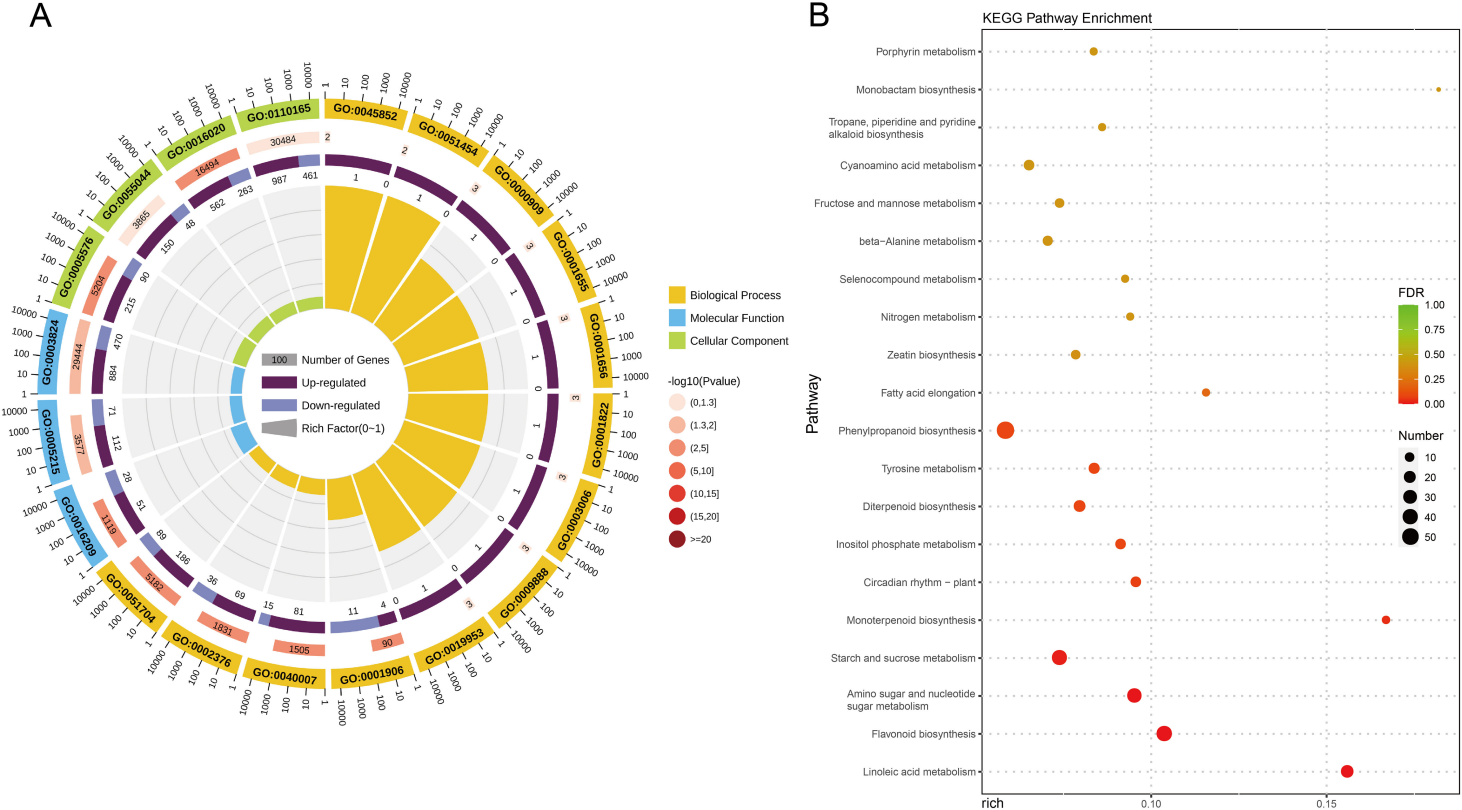
The functional annotation and analysis of DEGs in two comparison groups. **(A)** The top 20 enrichment pathway of DEGs by GO annotation. Complete data are presented in **Supplementary Table S12**. **(B)** The top 20 enrichment pathway of DEGs by KEGG. Complete data are presented in **Supplementary Table S13**. Color scale represents the gene expression level.

To analyze the differential expression patterns of the genes, differential expression analyses were conducted between the comparison groups. There were 2,301 DEGs between T5_12 and CK_12 (**Figure 3E**). To further investigate the potential roles of these DEGs, functional annotation and classification were performed using the GO annotation system (**Figure 4A**; **Supplementary Table S12**). The upregulated DEGs were mainly enriched for growth (GO:0040007), antioxidant activity (GO:0016209), extracellular region (GO:0005576), developmental process (GO:0003006), symplast (GO:0055044), multi-organism process (GO:0051704), membrane (GO:0016020), cellular anatomical entity (GO:0110165), cell junction (GO:0030054), immune system process (GO:0002376), and multicellular organismal process (GO:0051704). These functions suggest that they may be involved in the growth and development of seedlings after fertilization. In contrast, downregulated DEGs were significantly enriched in cell death (GO:0001906). Furthermore, KEGG enrichment analysis was performed to gain insight into the major pathways associated with the DEGs (**Figure 4B**; **Supplementary Table S13**). The upregulated DEGs were significantly enriched in numerous metabolic pathways including linoleic acid metabolism, terpenoid biosynthesis, phenylpropanoid biosynthesis, amino sugar and nucleotide sugar metabolism, fatty acid elongation, beta-alanine metabolism, zeatin biosynthesis, and nitrogen metabolism. However, flavonoid biosynthesis, starch and sucrose metabolism, and the circadian rhythm of plants were significantly downregulated. These results indicate that most of the DEGs were enriched in metabolic pathways related to the biosynthesis and metabolism of macromolecular compounds, including proteins, sugars, plant hormones, and secondary metabolites, potentially playing an important role in seedling growth.

Transcription factors (TFs), influenced by endogenous, developmental, and environmental inputs, play a vital role in the regulation of gene expression and plant development (Zhang et al., 2022). We identified 95 DEGs encoding TFs belonging to 22 families (**Figure 3D**). There were 66 upregulated and 29 downregulated genes after fertilization. The MYB family, with the highest number of TFs (25 genes), had 18 upregulated genes. All TFs from HD-ZIP, Trihelix, WRKY, ZF-HD, G2-like, and AP2 families were highly expressed. In contrast, the bHLH and NAC families had fewer upregulated TFs than the downregulated TFs.

### 3.5 Nutrient elements uptake and assimilation in response to NP fertilization

**Figure 5:**
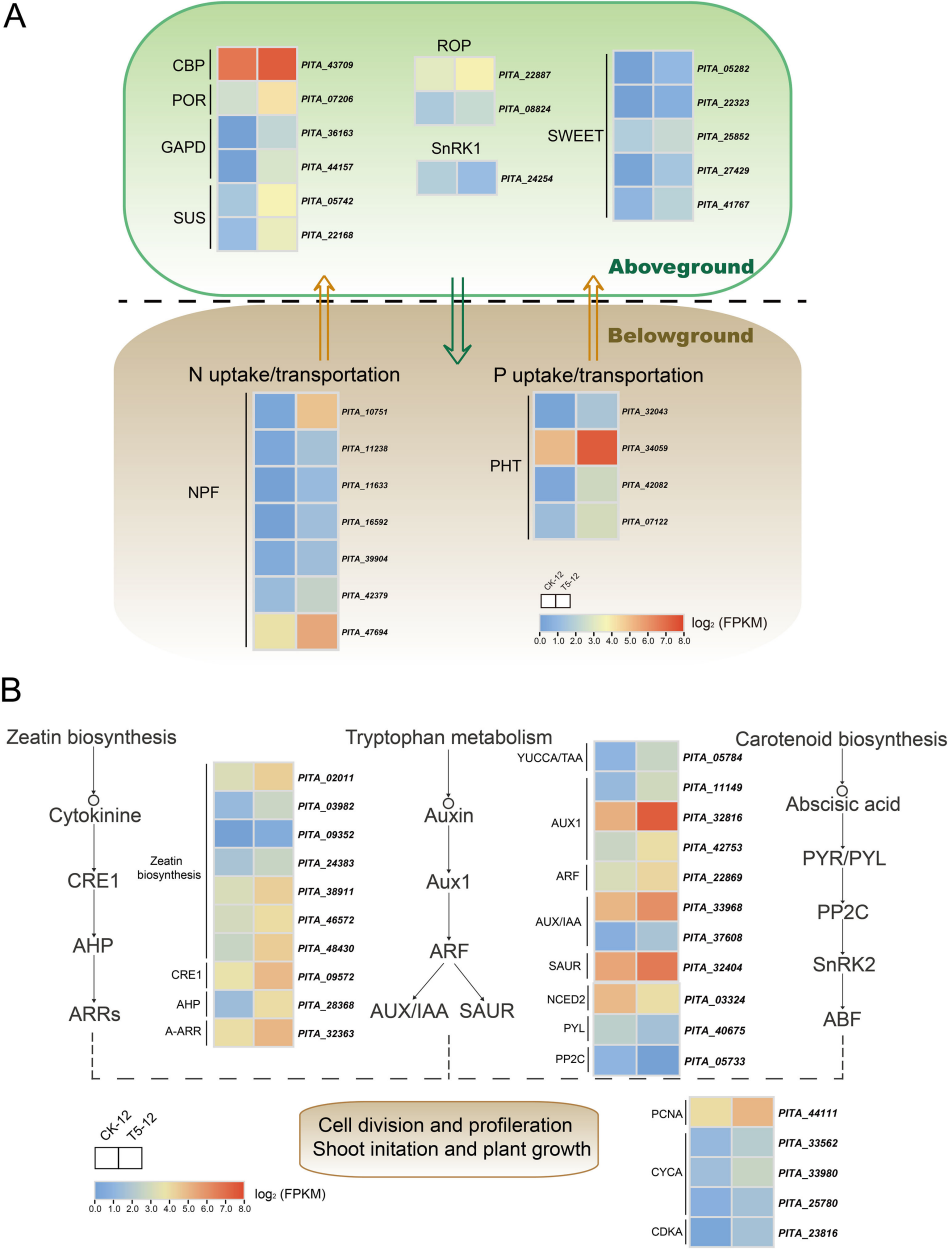
The expression profiles of DEGs associated with uptake and assimilation of nutrient under medium proportion of N and P fertilization. **(A)** The potential genes related to uptake and transportation of NP as well as photosynthesis. **(B)** The analysis of DEGs involved in phytohormones biosynthesis and signaling as well as cell proliferation. Complete data about DEGs are presented in **Supplementary Tables S14** and **S15**. Color scale represents the gene expression level.

The uptake and translocation of nutrient elements are closely related to the concentration, distribution, and use efficiency of nutrients in plants, and are often regarded as critical factors for improving plant growth and development (Daubresse et al., 2010; Xu et al., 2012; Wang et al., 2018). Therefore, we screened potential genes involved in the uptake and translocation of N and P and further investigated whether these genes exhibited transcriptional responses and were differentially expressed between the two treatments. In the current study, the differential expression of 11 genes was annotated in the NPF family, and four genes were related to the PHT family (**Figure 5A**). Among them, seven NPFs (*PITA_10751*, *PITA_16592*, *PITA_47694*, *PITA_11633*, *PITA_42379*, *PITA_11238*, and *PITA_39904*) and four PHTs (*PITA_42082*, *PITA_32043*, *PITA_07122*, and *PITA_34059*) were significantly upregulated. In particular, NPFs, which function as amphiphilic nitrate transporters, were specifically upregulated in response to N addition, with a 5.11-fold increase in *PITA_10751*. Additionally, *PITA_42082* and *PITA_32043*, which are responsible for PHT, were upregulated by 3.14- and 2.74-fold, respectively. These DEGs, which were specifically highly expressed in response to NP proportioning fertilization, could contribute to nutrient uptake and utilization in seedlings.

Photosynthesis, a critical process that regulates plant growth, is closely associated with chlorophyll synthesis and carbohydrate metabolism (Xu et al., 2012). Our current data show that leaf biomass and chlorophyll content increased significantly after fertilization. Therefore, we further identified potential key genes associated with these pathways (**Figure 5A**). We identified one *protochlorophyllide reductase* (POR) and one *chlorophyll a-b binding protein* (CBP) as pivotal genes for chlorophyll biosynthesis. Additionally, we found a number of DEGs enriched in the pathways of carbon fixation in photosynthetic organisms, as well as starch and sucrose metabolism. Among them, two *glyceraldehyde-3-phosphate dehydrogenase* genes (GAPD), responsible for key processes in the Calvin cycle and sugar metabolism, increased dramatically by 8.99- (*PITA_36163*) and 8.79-fold (*PITA_44157*), respectively. Two genes (*PITA_05742* and *PITA_22168*) annotated as *sucrose synthase* (SUS) and five genes (*PITA_41767*, *PITA_05282*, *PITA_22323*, *PITA_25852*, and *PITA_27429*) related to *bidirectional sugar transporter* genes (SWEET) were also highly upregulated. In contrast, the expression of *SNF1-related protein kinase* (SnRK1), specifically *PITA_24254*, significantly decreased by 1.74-fold under fertilizer supply conditions. The investigated genes, which regulate photosynthesis, accumulation, and transportation of photosynthetic products, were specifically expressed after fertilization. These expressions may be correlated with enhanced photosynthesis and may contribute to seedling growth.

### 3.6 Phytohormones and cell proliferation related genes in response to NP fertilization

Phytohormones play a crucial role in regulating plant development as well as enabling plants to sense and adapt to changing environments (Zhang et al., 2022). To confirm that the observed changes were not confined to genes related to nutrient element uptake and assimilation, the study also investigated the impact of NP application on the phytohormones regulation network and cell cycle. We screened for potential genes involved in the synthesis and signaling of phytohormones (**Figure 5B**). We found that 10 DEGs in CK biosynthesis and signaling pathways were upregulated under fertilization, including *histidine kinase/cytokinin receptor I* (CRE1), *histidine-containing phosphotransfer protein* (AHP), and *type A response factor* (A-ARR). Auxin biosynthesis, which is critical for modulating plant growth and differentiation, is closely correlated with tryptophan metabolism. We discovered that YUCCA/TAA, which encodes a pivotal enzyme for auxin synthesis, was specifically increased by 2.33-fold (*PITA_05784*) after fertilization. We also detected nine upregulated DEGs related to auxin signaling and responses. *Auxin response factors* (ARFs), which greatly contribute to the transcription of auxin-responsive genes (including Aux/IAA, GH3, and SAUR), were highly expressed. The abundance of Aux/IAA and SAUR increased with fertilization, whereas the expression of GH3 was not significantly different. Moreover, we found that genes involved in abscisic acid (ABA) biosynthesis and signaling, including *9-cis-epoxycatoteinoid dioxygenase* (NCED3), *abscisic acid receptor* (PYL), and *protein phosphatase 2C* (PP2C), were significantly downregulated compared to the control.

Furthermore, we investigated five genes involved in the cell cycle that were specifically upregulated in response to fertilization. These include one *proliferating cell nuclear antigen* (PCNA), three *cyclin A* (CYCA), and one *cyclin-dependent kinase* (CDKA). The expression of two DEGs (*PITA_22887* and *PITA_08824*) related to the *Rho GTPase-activating protein* (ROP) was higher than that in the unfertilized control. These screened genes, which are related to the biosynthesis and signaling of phytohormones, as well as the cell proliferation resulting from these processes, may synergistically regulate seedling growth.

### 3.7 Secondary metabolites related to biomass accumulation in response to NP fertilization

**Figure 6:**
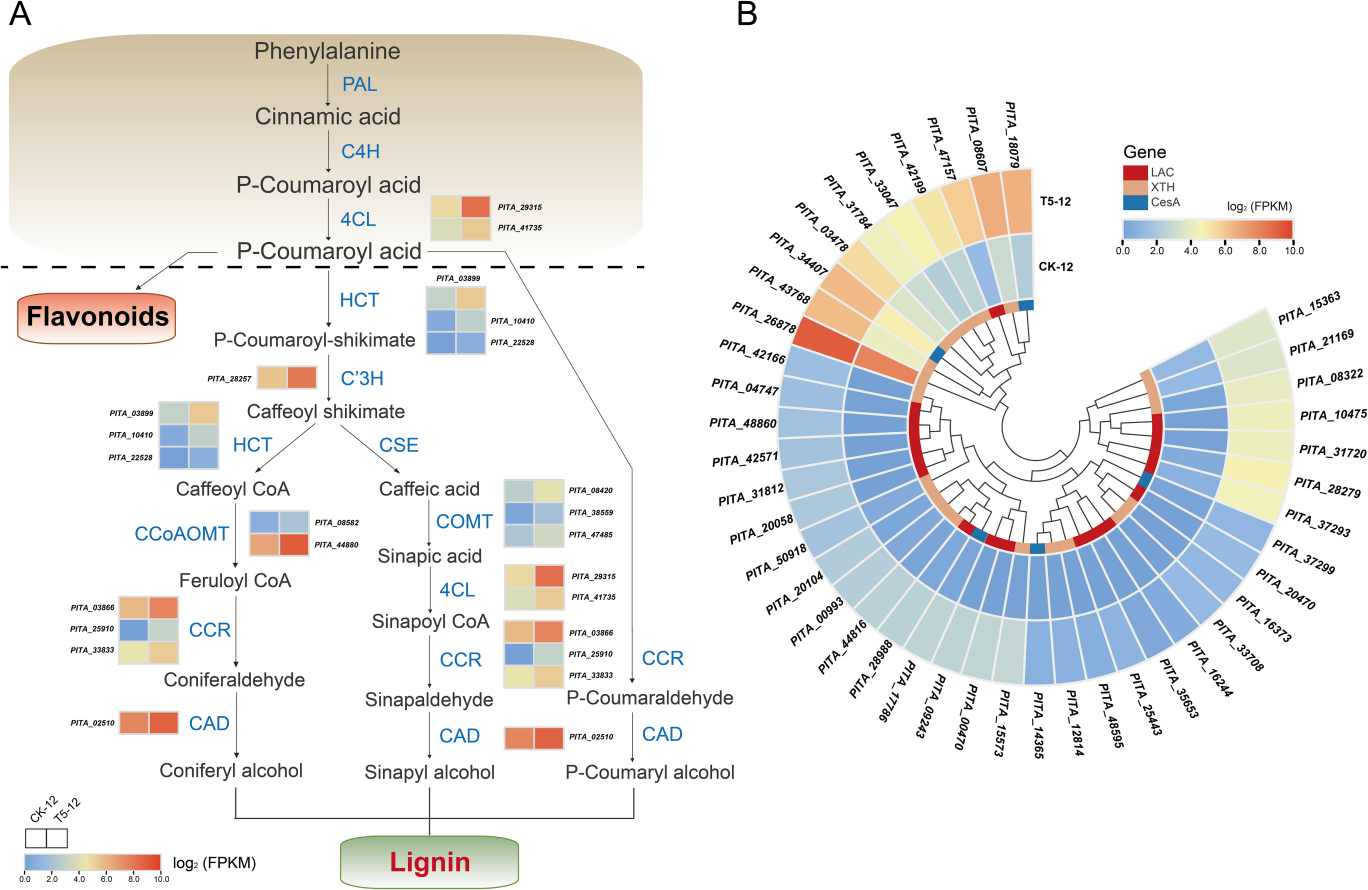
Effect of medium proportion of N and P fertilization on the biosynthesis of lignin and cellulose. **(A)** The pathway and related DEGs of phenylpropanoid biosynthesis in response to fertilization. **(B)** Heatmap analysis of DEGs potentially involved in the biosynthesis of lignin and cellulose. Complete data about DEGs are presented in **Supplementary Tables S14** and **S15**. Color scale represents the gene expression level.

Biomass increases significantly during seedling growth and development, primarily because of the biosynthesis of lignin and cellulose, which are the principal components of the plant skeleton (Dong and Lin, 2021). Our study focused on examining the potential key genes related to these pathways. Most lignin biosynthesis genes are closely correlated with the phenylpropanoid biosynthesis pathway. We found that 57 DEGs were involved in the phenylpropanoid biosynthesis pathway and lignin biosynthesis. Forty-three of these genes were upregulated expression, representing the majority (**Figure 6A**). Critical biosynthesis genes, such as *4-Coumarate-CoA ligase* (4CL), which provides precursors for lignin biosynthesis, were all upregulated under fertilization. The abundance of all DEGs was higher than that of the control. These include *shikimate O-hydroxycinnamoyltransferase* (HCT), *cinnamoyl-CoA reductase* (CCR), and *cinnamyl alcohol dehydrogenase* (CAD), which are essential for lignin catalysis and biosynthesis. Furthermore, monolignol biosynthesis genes, including *P-coumaroyl shikimate 3’-hydroxylase* (C3’H), *caffeoyl-CoA 3-O-methyltransferase* (CCoAOMT), and *Caffeate/5-hydroxyferulate 3-O-methyltransferase* (COMT), were also highly expressed during fertilization.

In addition, we detected twenty-two DEGs related to lignin and cellulose biosynthesis, including 17 *laccase* (LAC) genes and five *cellulose synthase A* (CesA) genes. These genes were specifically and dramatically upregulated in response to fertilization (**Figure 6B**). The *xyloglucan endotransglucosylase/hydrolase protein* (XTH), which regulates cell growth and differentiation during morphogenesis, is responsible for cell wall modification. Twenty genes encoding XTH were significantly upregulated compared with those in the control (**Figure 6B**). Thus, these results confirm the positive relationship between NP proportioning fertilization and the phenylpropanoid pathway and further provide molecular evidence for the role of lignin and cellulose biosynthesis in enhancing biomass accumulation during seedling growth.

### 3.8 Validation of RT-qPCR

**Figure 7:**
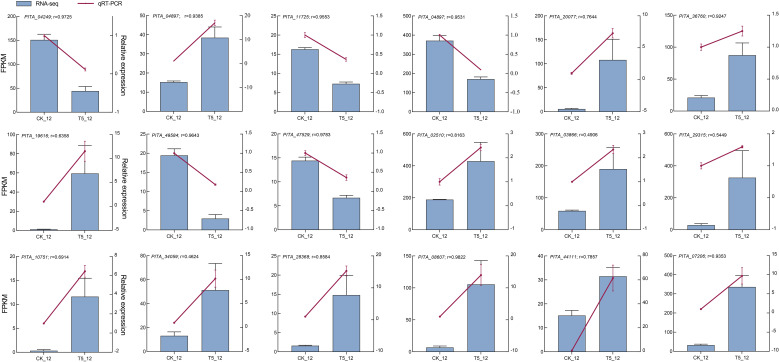
RT-qPCR verification of the expression profiles of 18 DEGs in seedlings. Histogram and left y-axis indicate FPKM values by RNA-seq, line charts, and right y-axis indicate the relative expression by RT-qPCR. The data are all mean normalized expression values ± standard error (SE) and determined by ANOVA analysis using GraphPad prism 9.5. The r indicates the Pearson’s correlation coefficient between RNA-seq and RT-qPCR.

Finally, to further confirm the transcriptome profiles, the relative expression levels of the 18 DEGs were assessed using RT-qPCR. Among them, nine genes (*PITA_04249*, *PITA_04879*, *PITA_11725*, *PITA_39609*, *PITA_20077*, *PITA_36760*, *PITA_19616*, *PITA_49584*, and *PITA_47529*) were randomly selected from a set of DEGs. Nine genes (*PITA_02510*, *PITA_03866*, *PITA_29315*, *PITA_10751*, *PITA_34059*, *PITA_28368*, *PITA_08607*, *PITA_44111*, and *PITA_07206*), which are likely involved in seedlings growth and development after NP proportioning fertilization, were selected for analysis. The results indicated that the expression patterns were similar to those obtained from the RNA-seq analysis. Furthermore, the average Pearson’s correlation coefficient was 0.814, suggesting reliability of the RNA-seq data and analysis (**Figure 7**).

## 4 Discussion

Fertilization plays an important role in promoting plant growth and enhancing crop productivity. Previous studies have reported that appropriate application of N and P in soil is critical to fostering seedling culture (Zhou et al., 2021; Yang et al., 2021; Luo et al., 2021). However, as a widespread and native silvicultural conifer in southwestern China; the appropriate fertilization dosage and proportion for *P. yunnanensis* seedlings have not been established, and the molecular mechanism in response to NP proportioning fertilization is largely unknown. In this study, we statistically analyzed the morphological, physiological, and biochemical characteristics of seedlings in response to different nutrient levels. The optimal fertilizer dosage and proportion during seedling growth were determined. Furthermore, the potential metabolic pathways and candidate genes that affect seedling growth in response to proportioning fertilization were identified (**Table 2**).

**Table 2:** FPKM values for DEGs involved in nutrients uptake, translocation, assimilation, and biomass accumulation in seedlings under medium proportion of N and P.

Gene id	Gene name	Function	Control (T1) ^1^	Treatment (T5)	log2 Fold Change
*PITA_10751*	NPF5;6	Nitrate uptake and translocation	0.33	11.61	5.11
*PITA_42082*	PHT1;5	Phosphate uptake and translocation	0.41	3.91	3.15
*PITA_43709*	CBP	Harvesting light and transmitting energy	11,595.84	28,916.47	1.27
*PITA_07206*	POR	Chlorophyll biosynthesis	32.81	335.83	3.31
*PITA_44157*	GAPD	Energy and intermediates metabolism	0.09	45.42	8.79
*PITA_05742*	SUS4	Sucrose synthesis and catabolism	7.57	182.31	4.52
*PITA_27429*	SWEET16	Transmembrane transport of sugars	0.17	6.19	5.06
*PITA_24254*	SnRK1	Energy sensing and nutrients metabolism	11.56	3.54	−1.76
*PITA_22887*	ROP5	Cell proliferation and growth	74.76	204.95	1.4
*PITA_08824*	ROP2	Cell proliferation and growth	8.58	23.35	1.38
*PITA_44111*	PCNA	Cell proliferation	15.16	31.51	1.02
*PITA_33562*	CYCA11	DNA replication and cell division	1.24	3.37	1.39
*PITA_33980*	CYCA22	DNA replication and cell division	1.78	5.27	1.54
*PITA_23816*	CDKA	DNA replication and cell division	0.2	2.05	3.37
*PITA_28368*	AHP1	Cytokinin signaling	1.59	14.8	3.19
*PITA_05784*	YUC9	Auxin biosynthesis	0.99	5.08	2.33
*PITA_32816*	AUX1	Auxin transport	35.39	182.01	2.29
*PITA_22869*	ARF4	Auxin signaling	7.85	17.12	1.06
*PITA_33968*	IAA13	Auxin signaling	31.48	67.24	1.04
*PITA_32404*	SAUR32	Auxin signaling	43.49	99.94	1.14
*PITA_03324*	NCED3	ABA biosynthesis	29.56	14.42	−1.08
*PITA_40675*	PP2C	ABA signaling	3.97	1.95	−1.08
*PITA_29315*	4CL	Monolignol biosynthesis and polymerization	29.43	326.41	3.4
*PITA_41735*	4CL	Monolignol biosynthesis and polymerization	12.37	42.17	1.72
*PITA_28257*	C3H	Monolignol biosynthesis and polymerization	45.7	257.6	2.42
*PITA_02510*	CAD	Monolignol biosynthesis and polymerization	188.54	429.38	1.12
*PITA_44880*	CCoAOMT	Monolignol biosynthesis and polymerization	91.14	503.91	2.39
*PITA_03866*	CCR1	Monolignol biosynthesis and polymerization	59.1	190.18	1.61
*PITA_08420*	COMT1	Monolignol biosynthesis and polymerization	5.58	17.2	1.57
*PITA_03899*	HCT	Monolignol biosynthesis and polymerization	6.76	42.36	2.58
*PITA_10475*	LAC11	Lignin polymers synthesis	0.12	18.22	7.15
*PITA_31720*	LAC17	Lignin polymers synthesis	0.07	17.16	7.73
*PITA_47157*	LAC6	Lignin polymers synthesis	1.47	56.05	5.19
*PITA_08322*	XTH32	Cell wall modification	0.24	15.36	5.93
*PITA_08607*	XTH8	Cell wall modification	6.76	105.56	3.91
*PITA_17786*	CESA9	Cellulose synthesis	0.22	4.98	4.46
*PITA_18079*	CESA4	Cellulose synthesis	3.74	99.72	4.67
*PITA_05699*	MYB46	Secondary cell wall and xylem development	1.36	16.92	3.56

^1^Note: the FPKM value was calculated by averaging of three biological replications.

### 4.1 NP proportioning fertilization regulate seedlings growth and biomass accumulation in *P. yunnanensis*

Appropriate fertilization can improve the seedling development. In woody plants, it has been found that height growth and aboveground development of seedlings are significantly improved by N (Zhou et al., 2021; Yang et al., 2021). When N and K are sufficient in the substrate, the application of P can effectively increase the basal diameter of *P. massoniana* seedlings and promote root growth (Luo et al., 2021). Our assays revealed that, as time progressed following fertilization, NP proportioning application promoted seedling growth more effectively than the single application of either N or P, as well as compared to treatments without fertilization. Specifically, we observed that high levels of N and P had a significant impact on seedling height, whereas high N combined with medium P significantly increased basal diameter.

The uptake of water and nutrients by roots is closely related to their morphological and physiological characteristics and influences the growth of the aerial parts of plants (Fan et al., 2015; Razaq et al., 2017). Plants can coordinate the growth of their aerial parts and roots and adapt to changing environments through biomass distribution (Bazzaz and Reekie, 2005). The proportion of biomass distribution among different organs exhibited significant differences at different stages after fertilization. The seedling biomass was preferentially distributed to the roots, averaging 36.71%, which may be correlated with the early adaptation strategy of seedlings by concentrating resources on roots to acquire nutrients (Cai et al., 2011). The priority of biomass distribution shifted from roots to aboveground parts as the developed roots promoted the growth of leaves and stems, with aboveground biomass averaging 83.27% at 300 d. This finding was consistent with the report of *Acer mono* (Razaq et al., 2017).

A study on *P. massoniana* found that NP proportioning fertilization promotes N uptake, which is related to biomass accumulation, while P mainly promotes root growth (Luo et al., 2021). The individual biomass of *T. amurensis* under medium NPK proportioning increased by 113.1% compared with that of the control (Yang et al., 2021). In our study, the biomass accumulation of stems, leaves, and individuals under medium proportions of NP surpassed that of the control (T1) and the groups receiving separate applications of N and P. Compared with T1, the roots, stems, leaves, and individuals of T5 increased by 63.8%, 38.6%, 39.2%, and 45.6% at 480 d, respectively. In contrast, the accumulation and distribution proportion of root biomass in T1 were higher than those in the other treatments before 210 d. This could be because the roots of unfertilized seedlings enhance nutrients acquisition by promoting root growth and distribution in the soil (Qin et al., 2019; Wang et al., 2022; Li et al., 2022).

### 4.2 NP proportioning fertilization regulate the content of nutrients and chlorophyll

Fertilization can influence the content and accumulation of nutrients in the seedlings (Nzokou and Cregg, 2010). The N, P, and K contents in plants affect photosynthesis and biomass accumulation (Montgomery, 2004). After proportioning fertilization, the accumulation of N in *P. massoniana* needles significantly increases (Luo et al., 2021). Here, we found that the reserves of N, P, and K in organs were higher after the medium proportion application of NP (T5) than after other treatments. Moreover, N, P, and K were primarily stored in the leaves, followed by roots and stems. Among these elements, N had the greatest content and reserve in organs, followed by K and P. Research on *Magnolia wufengensis* (Deng et al., 2019) and *Larix rupprechtii* (Zhao et al., 2014) indicate that leaves have higher nutrient content and reserves than other organs, such as assimilation and nutrient storage organs of seedlings. However, stems have fewer nutrient reserves because of their roles in transporting channels for nutrients. The total chlorophyll content peaked in all treatments at 210 d and coincided with the peak levels of N, P, and K in the leaves. This finding suggests that seedlings require sufficient N and K to enhance photosynthesis by increasing RuBisCO activity (Zhou et al., 2021). These results indicate that appropriate NP proportioning fertilization increases nutrient reserves and has a positive effect on promoting the accumulation of nutrients and further enhancing photosynthesis in seedlings.

### 4.3 NP proportioning fertilization regulate nutrients uptake and assimilation

Plant development mainly depends on their ability to acquire nutrients from the soil and transport and assimilate these nutrients (Daubresse et al., 2010). Inorganic N and P are absorbed and transported from the external environment by specific transmembrane proteins, such as NPFs and PHTs (Xu et al., 2012; Wang et al., 2018; Wei et al., 2023). We screened for related genes that were probably involved in the uptake and translocation of N and P and found that they were upregulated compared to unfertilized seedlings. Seven NPFs in our study increased by 1.17- to 5.11-fold, suggesting that they may have low and/or dual affinities for nitrate uptake and translocation (Wang et al., 2018). Many studies have reported that high-affinity PHTs are induced by P-stressed to promote P uptake (Ham et al., 2018; Wang et al., 2023). Our data indicate that several PHT genes responded to P application. The expression of low-affinity PHTs has been previously reported in *Arabidopsis* and rice. Under high P conditions, *OsPT1-OE* dramatically increased P content, whereas *OsPT1-RNAi* exhibited the opposite effect, suggesting that it functions as a low-affinity P transporter involved in the uptake and transport of P from the root to the shoot (Sun et al., 2012). Hence, we hypothesize that PHT genes induced by P application are low-affinity and/or dual affinity genes expressed in this study. How P starvation and sufficiency modulate the uptake and translocation of P in *P. yunnanensis* is a fascinating direction for future research.

Nutrient use efficiency depends not only on the uptake and translocation of plants but also on the availability of carbon provided by photosynthesis (Xu et al., 2012). CBP and POR, which are pivotal genes for light harvesting and chlorophyll biosynthesis (Ji et al., 2023), were upregulated during fertilization. Carbohydrates are crucial factors in seedling growth. The regulation of sucrose translocation from shoots to roots by *AtSWEET11* and *AtSWEET12* (Chen et al., 2012), two genes that were significantly upregulated by 2.64- and 4.64-fold in our study, may be involved in sucrose efflux from the phloem, promoting photosynthate translocation (Daubresse et al., 2010). The expression of SnRK1, a core regulator of plant energy-sensing deficiency, was significantly decreased by 1.74-fold under NP proportioning fertilization. This decrease was correlated with its function in coordinating the transcriptional regulatory network to maintain cellular energy homeostasis when energy supply is limited in *Arabidopsis* (Ramon et al., 2019). Based on these and previously measured biochemical data, we believe that the application of N and P facilitates the translocation and accumulation of nutrients and enhances photosynthetic efficiency.

### 4.4 NP proportioning fertilization regulate seedling growth by lignin biosynthesis-related genes

Lignin is the principal component of plant skeletons and maintains the growth of the roots and aerial parts (Guo et al., 2001). Monolignol biosynthesis and polymerization are major steps in lignin biosynthesis in the phenylpropanoid pathway (Dong and Lin, 2021). In *Betula platyphylla* (Hu et al., 2019) and *Malus domestica* (Chen et al., 2019), increased transcriptional abundance of C4H, 4CL, HCT, CCR, F5H, COMT, and CAD leads to lignin deposition and thickening of secondary cell walls. Here, both 4CLs were increased under fertilization, providing precursors for lignin biosynthesis in the general phenylpropanoid pathway. Notably, the abundance of HCTs during fertilization increased significantly. In *Arabidopsis*, the *AtHCT-RNAi* lines repress lignin biosynthesis while increasing flavonoid biosynthesis, highlighting the key function of HCT as a gateway enzyme during monolignol biosynthesis and polymerization (Besseau et al., 2007). In apples, MYB46 directly increases lignin deposition and secondary cell wall accumulation by binding to CAD, COMT and CCR promoters (Chen et al., 2019). Interestingly, our transcript analysis also revealed the upregulation of MYB46 expression (**Supplementary Table S15**). The crucial reaction enzyme (CCR) and final catalyzed step (CAD) in lignin catalysis and biosynthesis were expressed at higher levels than those in the control. Furthermore, we observed upregulation of 17 LACs and five CesAs, which are involved in the biosynthesis of lignin and cellulose, respectively, in response to fertilization. The abundance of XTHs was significantly higher than that in the control, which could be correlated with an increase in cell volume and cell wall synthesis during the physiological process of seedling growth regulation (Xu et al., 2020). These data suggest that the application of NP can contribute to the regulation of cell wall formation, lignin biosynthesis, and wood formation, improving seedling growth and increasing the biomass of organs (Dong and Lin, 2021).

### 4.5 Phytohormone regulation and cell proliferation response to NP fertilization

The biosynthesis and signaling of endogenous phytohormones in plants are affected by nutrients (Xu et al., 2012). Here, we revealed that most of the highly expressed DEGs in zeatin biosynthesis stimulated the CK receptor CRE1, which further activated AHP expression. This finding aligns with previous reports, indicating that N uptake promotes CK synthesis in the shoot and stimulates cell division, thereby promoting the development of *Arabidopsis* and rice (Kieber and Schaller, 2018). Moreover, genes involved in cell division and proliferation, including PCNA, CYCA, and CDKA, were significantly upregulated. They are essential for meristem activity and bud outgrowth (Kotov et al., 2021). Auxin biosynthesis is closely correlated with modulation of plant growth and differentiation. In *Arabidopsis*, nitrate promotes auxin accumulation by repressing NFP6;3 expression (Wang et al., 2018). We found that YUCCA/TAA, which encodes a pivotal synthetase for auxin, was upregulated after fertilization. In addition, ARFs and their downstream genes, including Aux/IAA and SAUR, were highly expressed. Aux/IAA can be directly induced by nitrate and *AtNRT1;1*, and plays a role in transporting auxin from shoots to roots (Xu et al., 2012). Our results are consistent with the typical pathway involving ARF–SAUR–PM H + ATPase, which facilitates cell elongation and plant growth (Leyser, 2018). ABA has been extensively studied and utilized as a major phytohormone that plays a role in inhibiting bud outgrowth and seedling development (Kotov et al., 2021). In response to fertilization, the ABA biosynthesis gene NCED3 and signaling gene PP2C were significantly downregulated, suggesting that fertilization promoted seedling growth by inhibiting the expression of ABA-related genes. Furthermore, the ROP-TOR pathway can integrate diverse N and hormone signals to promote cell proliferation and growth in *Arabidopsis* and apples (Liu et al., 2021; Li et al., 2022). We observed that the transcript abundance of ROP was significantly upregulated. Hence, we consider that after NP proportioning fertilization, seedling growth may be promoted through the crosstalk between nutrient elements and phytohormones.

## 5 Conclusion

**Figure 8:**
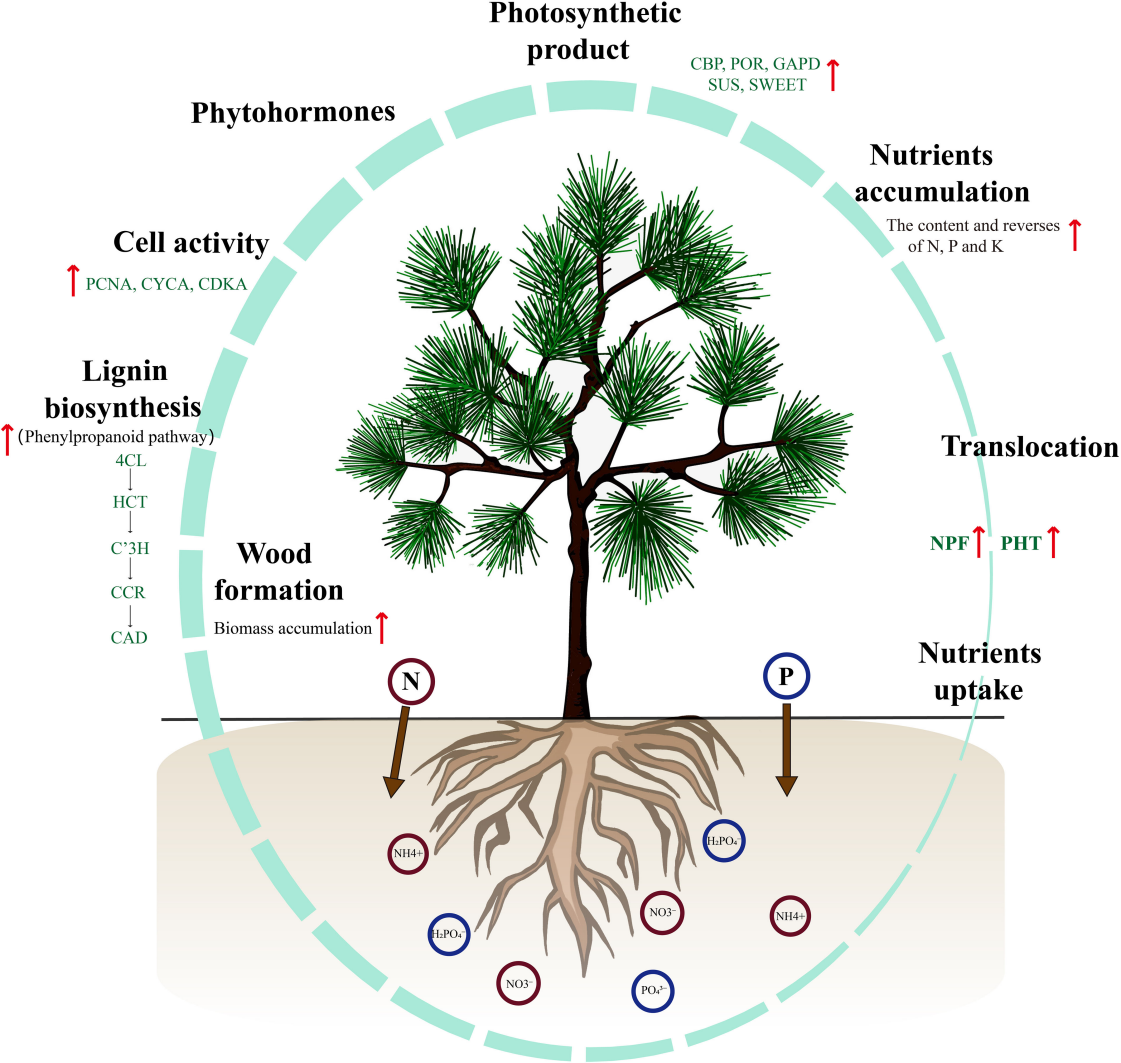
A schematic model represents the growth of *P. yunnanensis* seedlings when application of N and P proportion. During the application of N and P proportions, NPFs and PHTs regulate nutrient uptake and translocation activity to promote root growth and biomass accumulation. With seedling development, the content of total chlorophyll, nitrogen, phosphorus, and potassium gradually increase and reach peaks at 210 d after fertilization. Subsequently, the specific expression of genes involved in photosynthesis, assimilation, and transport of photosynthetic products facilitate significantly increase in seedlings biomass. These processes-associated genes are mainly involved in the phytohormones biosynthesis and signaling, cell cycle regulation, and lignin biosynthesis.

In this study, we propose a working model that provides comprehensive insights into the morphological, physiological, biochemical, and transcriptomic changes that occur in response to the application of NP proportioning in *P. yunnanensis* seedlings (**Figure 8**). The growth rate, biomass accumulation, photosynthetic rate, and nutrient levels of seedlings were significantly affected by N and P proportioning fertilization. In particular, under medium N and P conditions, the biomass accumulation rate of the seedlings was significantly higher than that of the others. The analysis of nutrient elements suggests that T5 (nitrogen 0.4 g·per^−1^, phosphorus 3 g·per^−1^) has higher N and P utilization efficiency. These results provide a foundation for using optimal levels of N and P to enhance nutrient use efficiency and shorten breeding cycles in *P. yunnanensis* afforestation practices. It is also important to increase plantation productivity. Furthermore, under N and P proportioning conditions, the upregulated expression of genes related to nutrient uptake, translocation, and assimilation influenced the accumulation and utilization of nutrients in seedlings. Lignin biosynthesis and its related genes significantly affected biomass accumulation in seedlings. These findings provide new insights into the relationship between nutrient utilization and development in *P. yunnanensis* seedlings. Future research should investigate the response and functional mechanisms of NPF and PHT genes to varying nutrient concentrations, which is a valuable direction for investigation. Additionally, clarifying how phytohormones interact with N and P to regulate nutrient assimilation and plant development is a fascinating direction for further research.

## Data Availability

The datasets presented in this study can be found in online repositories. The names of the repository/repositories and accession number(s) can be found in the article/**Supplementary Material**.
