# Supplementary material for: Integrative analyses of morpho-physiological, biochemical, and transcriptomic reveal the seedling growth response of Pinus yunnanensis to nitrogen and phosphorus fertilization
Source: Front Plant Sci. 2025 Jan 27;15:1405638. doi: 10.3389/fpls.2024.1405638 (PMC11807977; doi:10.3389/fpls.2024.1405638)
Supplement: Supplementary file 1 [file SupplementaryFile1.docx]

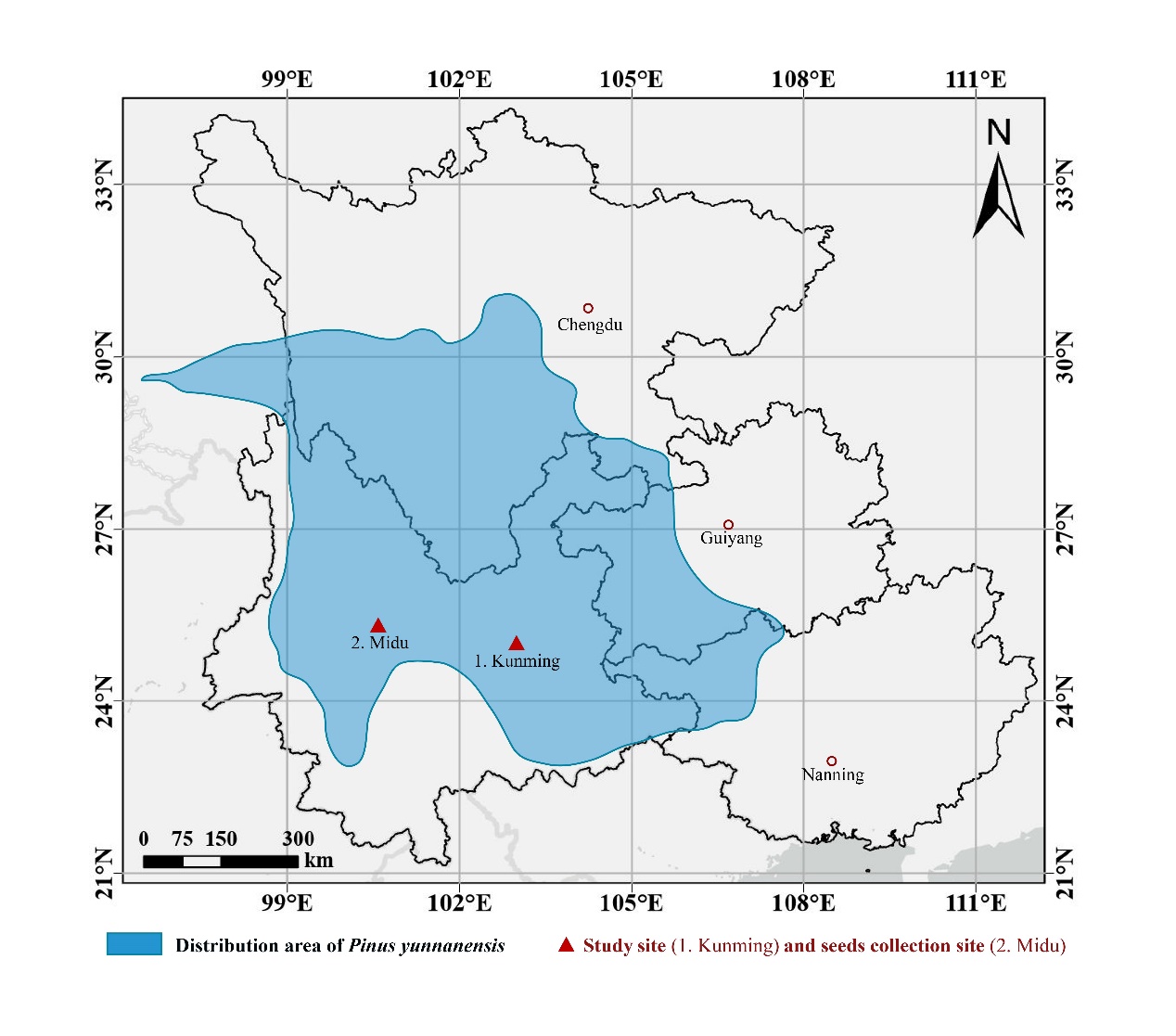


**Supplementary Figure 1.** The schematic map of the natural distribution area of *Pinus yunnanensis*, as well as the seeds collection and research locations for this study. The source and drawing of the geographical map were conducted using ArcGIS 10.6 (<https://doc.arcgis.com/en/archive/>). The area within the blue outline represents the natural distribution area of *P. yunnanensis*, and the red triangle represents the sites of seeds collection and research, respectively, for this study. The seeds were collected from a clonal seed orchard in Midu County, Yunnan Province, Dali, China. The experimental field was set up at an open nursery garden located at Southwest Forestry University, Yunnan Province, Kunming, China.


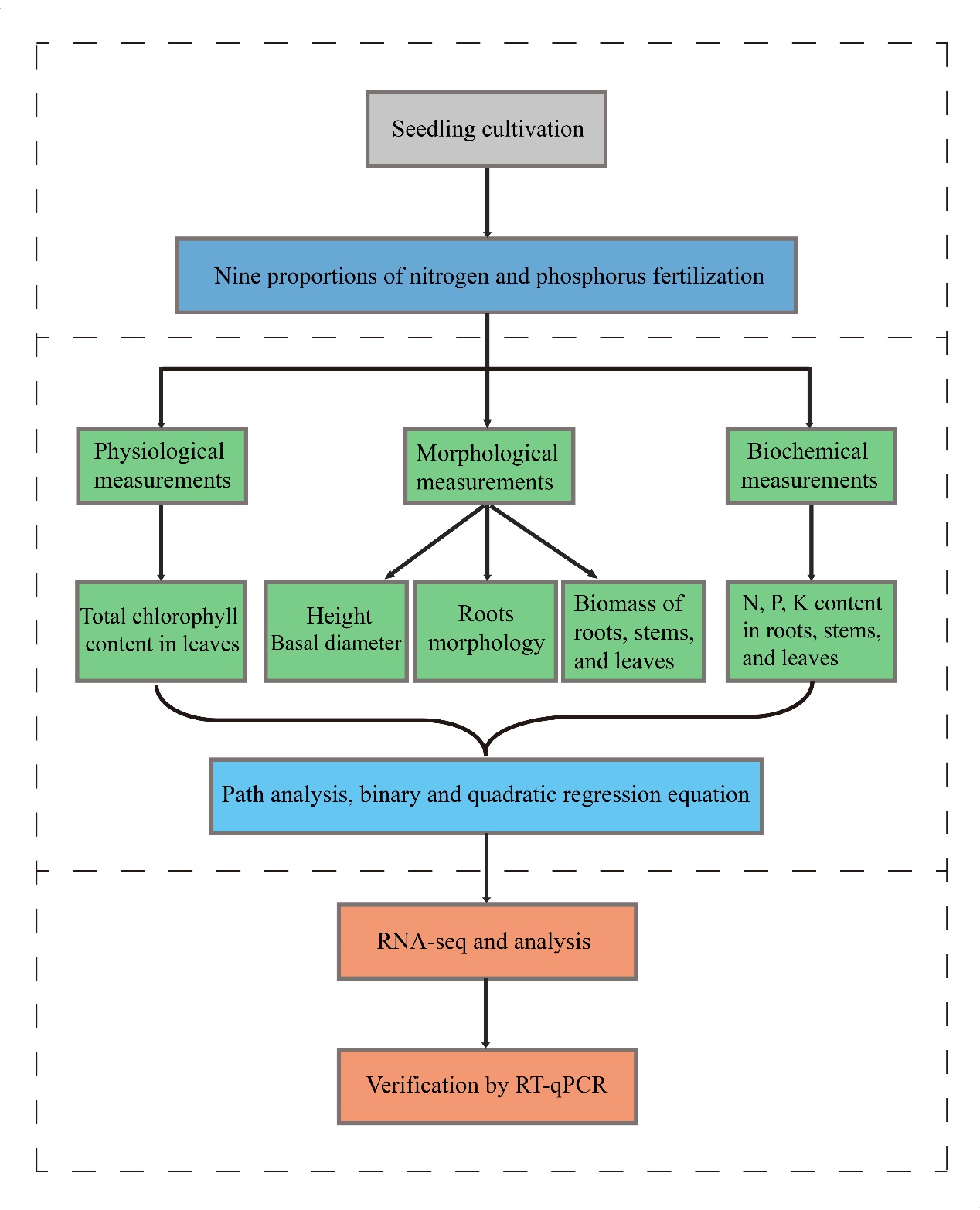


**Supplementary Figure 2.** Overview of the experiment workflow. This study conducted phenotypic, physiological, and biochemical measurements on *P. yunnanensis* seedlings after applying nine different proportions of nitrogen (N) and phosphorus (P), and utilized transcriptome analysis to reveal the effects of N and P proportioning application on the seedlings growth.
